# Analysis of urinary oligosaccharides in lysosomal storage disorders by capillary high-performance anion-exchange chromatography–mass spectrometry

**DOI:** 10.1007/s00216-012-5968-9

**Published:** 2012-04-20

**Authors:** Cees Bruggink, Ben J. H. M. Poorthuis, André M. Deelder, Manfred Wuhrer

**Affiliations:** 1 Biomolecular Mass Spectrometry Unit, Department of Parasitology, Leiden University Medical Center, P.O. Box 9600, 2300 RC Leiden, The Netherlands; 2 Department of Medical Biochemistry, Academic Medical Center, Meibergdreef 15, 1105 AZ Amsterdam, The Netherlands; 3 Thermo Fisher Scientific, Abberdaan 114, 1046 AA Amsterdam, The Netherlands

**Keywords:** HPAEC-IPAD, Catabolism, Metabolic disorder, Clinical glycomics, *N*-linked glycans, Glycolipids

## Abstract

Many lysosomal storage diseases are characterized by an increased urinary excretion of glycoconjugates and oligosaccharides that are characteristic for the underlying enzymatic defect. Here, we have used capillary high-performance anion-exchange chromatography (HPAEC) hyphenated to mass spectrometry to analyze free oligosaccharides from urine samples of patients suffering from the lysosomal storage disorders fucosidosis, α-mannosidosis, G_M1_-gangliosidosis, G_M2_-gangliosidosis, and sialidosis. Glycan fingerprints were registered, and the patterns of accumulated oligosaccharides were found to reflect the specific blockages of the catabolic pathway. Our analytical approach allowed structural analysis of the excreted oligosaccharides and revealed several previously unpublished oligosaccharides. In conclusion, using online coupling of HPAEC with mass spectrometric detection, our study provides characteristic urinary oligosaccharide fingerprints with diagnostic potential for lysosomal storage disorders.

**Electronic supplementary material:**

The online version of this article (doi:10.1007/s00216-012-5968-9) contains supplementary material, which is available to authorized users.

## Introduction

Fucosidosis, α-mannosidosis, G_M1_-gangliosidosis, G_M2_-gangliosidosis, and sialidosis are autosomal recessive lysosomal storage diseases (LSD). These LSDs are the result of defects of one or more enzymes or cofactors involved in the catabolism of glycoconjugates that takes place in the lysosome. Fucosidosis is caused by a deficient lysosomal α-l-fucosidase (EC 3.2.1.51) and results in secretion of fucosyl-oligosaccharides [1, 2]. Deficient lysosomal α-d-mannosidase (EC 3.2.1.24) causes α-mannosidosis and excessive urinary excretion of oligomannosidic glycans [3–5]. Sialidosis is caused by deficient acid exo-α-sialidase (EC 3.2.1.18) [6]. The urinary excretion of sialyloligosaccharides is similar to that found in galactosialidosis [1, 7]. G_M1_-gangliosidosis is a neurosomatic disease due to the deficient activity of β-galactosidase (EC 3.2.1.23) [8, 9]. In addition to the storage of G_M1_-gangliosides, glycoconjugates with β-galactose at the non-reducing end are increased in patients’ urine.

G_M2_-gangliosidosis is a group of three disorders (1) Tay-Sachs disease, (2) Sandhoff disease, and (3) AB variant. For all variants of G_M2_-gangliosidosis, the major neural storage compound is ganglioside G_M2_ [10–12]. Only in Sandhoff disease oligosaccharides derived from glycoproteins accumulate due to the deficiency of β-hexosaminidase A in addition to the (functional) deficiency of β-hexosaminidase B [13]. Blockage of the *N*-glycan catabolism results in accumulation of oligosaccharides carrying a single *N*-acetylglucosamine residue at the non-reducing end in tissues and urine of Sandhoff disease patients [14–16]. The current study includes the analysis of urine samples of patients suffering from Sandhoff disease.

Biochemical screening of these LSDs is usually performed using thin-layer chromatography (TLC) [17–19], since TLC is relatively easy to perform and does not require expensive equipment. However, interpretation of a TLC pattern of excreted oligosaccharides requires much experience in pattern recognition. On the other hand, liquid chromatography combined with UV [20] or fluorescence [21] detection is easier to reproduce and to interpret [22, 23].

Hyphenation of liquid chromatography with mass spectrometry allows the detailed characterization of oligosaccharides [24]. We have previously described a capillary high-performance anion-exchange chromatograph (HPAEC) setup with combined integrated pulsed amperometric (IPAD) and ion trap mass spectrometric detection which was used to characterize oligosaccharides from urine of G_M1_-gangliosidosis [25] and galactosialidosis [26] patients. This combination of chromatography IPAD and mass spectrometric detection allows detailed glycan analysis and characterization, when compared with TLC, HPLC, or HPAEC-IPAD without mass spectrometry (MS). Using this analytical setup, we report on the analysis of oligosaccharides in urine samples of fucosidosis, α-mannosidosis, G_M1_-gangliosidosis, G_M2_-gangliosidosis, and sialidosis patients. The results provided in glycan fingerprints that are found to be characteristic for the individual diseases and reflect the specific enzymatic defects.

## Materials and methods

### Materials

Analytical-reagent-grade sodium hydroxide (50% *w/w*), sodium acetate, sulfuric acid, and sodium chloride were obtained from J.T. Baker (Deventer, The Netherlands). Acetonitrile was from Biosolve (Valkenswaard, The Netherlands). All solutions were prepared using water from a Milli-Q synthesis system from Millipore BV (Amsterdam, The Netherlands). Details on urine samples are given in Table 1.

**Table 1:** Information about the urine samples

Sample code	Disorder	Sex	Age (years)	Creatinine (mmol/L)
U1	Fucosidosis	M	18	2.22
U2	G_M1_-gangliosidosis	F	0.42	1.04
U3	G_M2_-gangliosidosis	M	0.75	5.37
U4	G_M2_-gangliosidosis	M	0.58	1.04
U5	α-Mannosidosis	M	22	18.86
U6	α-Mannosidosis	F	7	8.46
U7	α-Mannosidosis	M	20	14.52
U8	Sialidosis	F	Unknown	Not determined

The age at the time point of sample gathering is given

### Sample preparation

Oligosaccharides of the samples were isolated with graphitized carbon solid-phase extraction, as described previously [27]. A 200-μL sample was diluted with 1,800 μL water and loaded on a Carbograph SPE (210142) from Alltech Associates Inc. (Deerfield, IL). The cartridge was washed with 6 mL of demineralized water. The oligosaccharides were eluted from the column with 3 mL of 25% acetonitrile containing 0.05% trifluoroacetic acid. The eluate was evaporated under a nitrogen stream at room temperature until the volume was decreased by 50%. The remaining solution was lyophilized and reconstituted with 200 μL water.

### Capillary HPAEC

The capillary chromatographic system consists of a modified Dionex BioLC system from Thermo Fisher Scientific (Sunnyvale, CA, USA) comprising a microbore GP40 gradient pump, a Famos micro-autosampler with a full PEEK (polyether ether ketone) injector equipped with a 1 μL loop and an ED40 electrochemical detector. BioLC control, data acquisition from the ED40 detector, and signal integration was supported by Dionex Chromeleon software (Themo Fisher Scientific). This modified system has been described in detail before [25]. A prototype capillary column (250 × 0.4 mm I.D.) packed with CarboPac PA200 resin was manufactured by Thermo Fisher Scientific. The GP40 eluent flow was split by a homemade PEEK splitter to 10 μL min^−1^. The pump was provided with the following eluents: eluent A, water; eluent B, 500 mM sodium hydroxide; eluent C, 500 mM sodium acetate. All separations were performed at room temperature. The following ternary gradient was used for separating oligosaccharides of fucosidosis, G_M2_-gangliosidosis, and sialidosis—76% A + 24% B (−20 to −14 min) isocratic sodium hydroxide column wash; 88% A + 12% B (−14 to 0 min) isocratic equilibration of the column; a linear sodium acetate gradient (0–55 min) to 25.5% A + 12% B + 62.5% C was used for the separation. For separating oligosaccharides of α-mannosidosis and G_M1_-gangliosidosis, the following ternary gradient was used—76% A + 24% B (−20 to −14 min) isocratic sodium hydroxide column wash; 88% A + 12% B (−14 to 0 min) isocratic equilibration of the column; linear sodium hydroxide gradient (0 to 9.1 min) to 60% A + 40% B; 60% A + 40% B (9.1 to 12.5 min) isocratic; linear gradient (12.5 to 21.6 min) to 85.2% A + 12% B + 2.8% C; linear sodium acetate gradient (21.6 to 104 min) to 60.5% A + 12% B + 27.5% C. Samples were injected at time 0.0 min.

The ED40 detector applies the following waveform to the electrochemical cell—*E*_1_ = 0.1 V (*t*_d_ = 0.00–0.20 s, *t*_1_ = 0.20–0.40 s), *E*_2_ = –2.0 V (*t*_2_ = 0.41–0.42 s), *E*_3_ = 0.6 V (*t*_3_ = 0.43 s), *E*_4_ = –0.1 V (*t*_4_ = 0.44–0.50 s) versus an Ag/AgCl reference electrode [28]. A 1 mm gold work electrode and a 25 μm gasket were installed.

### Mass spectrometry

Coupled to the chromatographic system was an Esquire 3000 ion trap mass spectrometer from Bruker Daltonics (Bremen, Germany), equipped with an electrospray ionization source. To convert the HPAEC eluate into an ESI compatible solution, an in-line prototype desalter (Thermo Fisher Scientific) was used which was continuously regenerated with 12.5 mM sulfuric acid at a flow rate of 0.8 mL min^−1^ [25]. A modified microbore AGP-1 (Thermo Fisher Scientific) was used as an auxiliary pump: To obtain efficient ionization of the eluted carbohydrates in the positive mode, 0.6 mM NaCl in 50% acetonitrile was pumped into the eluent flow via a MicroTEE (P-775 Upchurch Scientific, Oak Harbor, WA, USA) at a flow rate of 4.6 μL min^−1^. The mixture was directed to the electrospray ionization interface of the Esquire 3000 used in the positive mode. The MS was operated at the following conditions: dry temperature 325 °C, nebulizer 103 kPa, dry gas 7 l min^−1^, capillary voltage −3,500 V, target mass *m/z* 850, scan speed 13,000 *m/z*/ s in MS mode, scan range 150–2,000 *m/z*, ICC target 50,000 with maximum accumulation time 50 ms. For tandem MS, automatic selection of three precursors was applied with absolute intensity threshold 10,000 and 5% relative intensity threshold (relative to the base peak intensity), using fragmentation settings of 1.40 V with smart fragmentation amplification of 30%–100% and a fragmentation time of 40 ms.

### System suitability check

To check the correct functioning of the complete instrumental setup, every sequence started with analyzing a 50 nmol mL^−1^ lactose solution with 60 mM NaOH as eluent. The resulting MS chromatogram should pass the following criteria: The retention time has to be 7.5 min ± 15%; in the total ion current chromatogram, the baseline level intensity should be ≤4.5 e6 with the noise intensity ≤7.5 e5; for the extracted ion chromatogram (*m/z* 365 ± 0.5), the peak height intensity ≥4.5 e6 with a peak width at half height of ≤55 s.

### Data analysis

MS as well as MS/MS spectra were manually interpreted using DataAnalysis (version 3.3, Bruker Daltonics). The extracted ion chromatograms (EIC) were used in order to determine the peak area of oligosaccharides present in the MS spectra. Signals of all detected charge states and isomers corresponding to the same compound were added up. Peak areas were normalized to the sum of all glycan peak areas of one sample.

## Results

Free oligosaccharides from eight urine samples of patients suffering from various LSDs including fucosidosis, α-mannosidosis, G_M1_-gangliosidosis, G_M2_-gangliosidosis, and sialidosis (Table 1) were analyzed by HPAEC-IPAD-MS to investigate disease-related, excreted degradation products. A total of 54 glycans were analyzed in these urine samples as sodium adducts using positive ion mode mass spectrometry. The set of 54 glycans was established by manual assignment of glycan species from all HPAEC-MS(/MS) data. This set includes glycans described previously in literature for the LSDs included in this study [2, 9, 15, 16, 25, 29–40] as well as the glycans found previously for galactosialidosis samples [26].

### Urinary glycans in fucosidosis

**Fig. 1:**
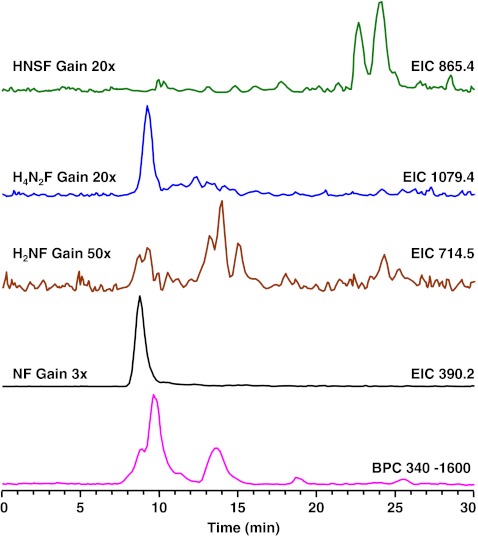
Separation of oligosaccharides in urine of a fucosidosis patient. *H* hexose, *N N*-acetylhexosamine, *F* fucose, *S N*-acetylneuraminic acid, *BPC* base peak chromatogram

**Fig. 2:**
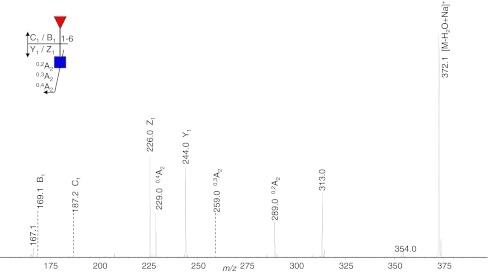
Positive-ion fragmentation mass spectrum of the monosodiated disaccharide HexNAc_1_Fuc_1_ (precursor ion at *m/z* 390.2) from urine of a fucosidosis patient. *Red triangle* = fucose, *blue square* = *N*-acetylglucosamine

**Fig. 3:**
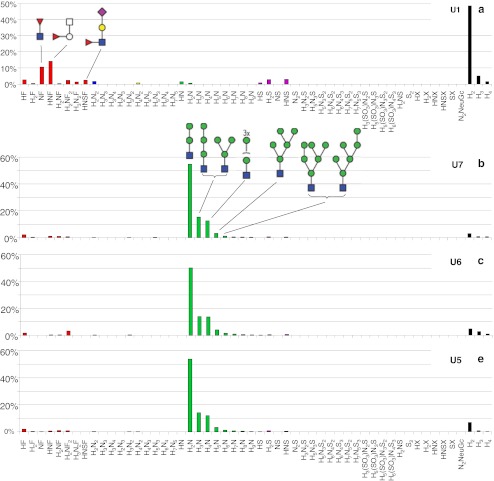

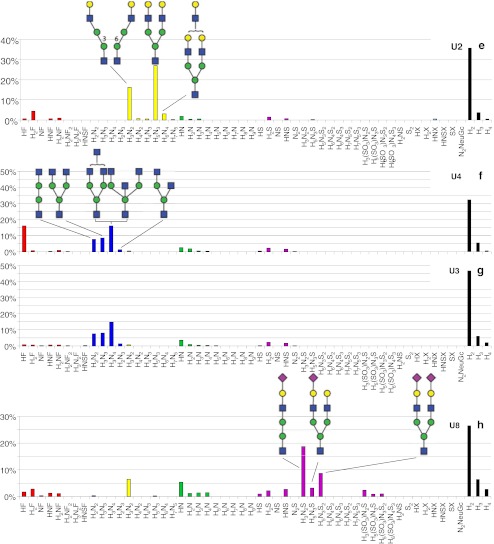
Histograms showing the relative abundance of the detected glycans in the urine sample (Table 1) of lysosomal storage disorders fucosidosis (**a**), α-mannosidosis (**b**, **c**, and **d**), G_M1_-gangliosidosis (**e**), G_M2_-gangliosidosis (**f** and **g**), and sialidosis (**h**). *H* or *white circle* = hexose, *N* or *white square* = *N*-acetylhexosamine, *F* or *red triangle* = fucose, *S* or *purple diamond* = *N*-acetylneuraminic acid, NeuGc = *N*-glycolylneuraminic acid, *X* = hexonic acid, *SO*_*3*_ = sulfate, *yellow circle* = galactose, *green circle* = mannose, *blue square* = *N*-acetylglucosamine

Eight fucosylated oligosaccharides were detected in the urine sample of a fucosidosis patient, and the EIC of four of these fucosyl oligosaccharides are shown in Fig. 1. The neutral, fucosylated oligosaccharides were observed in an early retention time window (7 to 15 min), while the acidic species HNSF resulted in signals between 22 and 25 min. In order to enable relative quantification of the oligosaccharides, the signals were normalized to the overall intensity of detected MS signals. Glycan species were characterized by tandem mass spectrometry as exemplified for the fucosyl disaccharide Fuc-HexNAc which had a relative abundance of 10.6% (Table 2; Fig. 2). The MS/MS fragmentation spectrum (Fig. 2) showed Z_1_ and B_1_ fragments as well as a prominent signal arising from the loss of water (*m/z* 372.1). Cross-ring cleavages at *m/z* 229.0 (^0,4^A_2_), 259.0 (^0,3^A_2_), and 289.0 (^0,2^A_2_) suggest a 1–6 linkage for the fucose residue [41–43]. From the total set of 54 glycan compositions observed in this study, 17 were found to be present in the fucosidosis sample (see Electronic supplementary material Table S1) resulting in a glycan fingerprint as shown in Fig. 3a. Of the eight fucosylated oligosaccharides detected, three have already been previously shown to be related to fucosidosis [2, 29, 30, 37, 44].

**Table 2:** Oligosaccharide species detected in urine samples U1–U8 and their relative area

Comp.	Registered *m/z*	Charge state	Relative area							
			Fuc.	G_M1_-gang.	G_M2_-gangliosidosis		α-Mannosidosis			Sial.
			U1	U2	U3	U4	U5	U6	U7	U8
HF	349.2	[M + Na]^+^	2.6%	0.6%	0.7%	16.0%	1.9%	1.6%	2.1%	1.7%
H_2_F	511.3	[M + Na]^+^	0.3%	4.4%	0.5%	0.7%	0.2%	0.0%	0.3%	2.8%
NF	390.2	[M + Na]^+^	10.6%	0.0%	0.2%	0.0%	0.1%	0.0%	0.0%	0.3%
HNF	552.5	[M + Na]^+^	14.1%	0.6%	0.5%	0.2%	0.6%	0.2%	1.2%	1.2%
H_2_NF	714.5	[M + Na]^+^	0.3%	0.9%	0.6%	0.8%	0.9%	0.3%	1.0%	1.1%
H_2_NF_2_	860.5	[M + Na]^+^	2.3%	0.0%	0.2%	0.1%	0.7%	3.2%	0.7%	0.0%
H_3_N_2_F	1079.4	[M + Na]^+^	1.3%	0.0%	0.0%	0.0%	0.0%	0.0%	0.0%	0.0%
HNSF	865.4	[M–H + 2Na]^+^	2.3%	0.0%	0.3%	0.0%	0.0%	0.0%	0.0%	0.0%
H_2_N_2_	771.5	[M + Na]^+^	1.6%	0.0%	7.6%	7.6%	0.2%	0.1%	0.1%	0.3%
H_3_N_2_	933.5; 478.3	[M + Na]^+^; [M + 2Na]^2+^	0.0%	16.2%	0.6%	0.4%	0.1%	0.2%	0.1%	6.5%
H_4_N_2_	1095.5	[M + Na]^+^	0.7%	0.7%	0.0%	0.0%	0.1%	0.0%	0.0%	0.0%
H_4_N_3_	1298.5; 660.9	[M + Na]^+^; [M + 2Na]^2+^	0.0%	0.5%	0.0%	0.0%	0.0%	0.0%	0.0%	0.0%
H_3_N_3_	1136.5; 580.0	[M + Na]^+^; [M + 2Na]^2+^	0.0%	0.0%	8.0%	8.2%	0.0%	0.0%	0.0%	0.0%
H_3_N_4_	1339.4; 681.2	[M + Na]^+^; [M + 2Na]^2+^	0.0%	0.0%	14.8%	15.5%	0.0%	0.0%	0.0%	0.0%
H_2_N_3_	974.6	[M + Na]^+^	0.0%	0.0%	1.3%	1.2%	0.0%	0.0%	0.0%	0.0%
H_5_N_3_	1460.6; 742.1	[M + Na]^+^; [M + 2Na]^2+^	0.0%	27.1%	0.0%	0.0%	0.0%	0.0%	0.2%	0.3%
H_6_N_4_	924.5	[M + Na]^+^	0.0%	3.0%	0.0%	0.0%	0.0%	0.0%	0.0%	0.0%
H_7_N_5_	1107.0	[M + 2Na]^2+^	0.0%	0.3%	0.0%	0.0%	0.0%	0.0%	0.0%	0.0%
HN	406.2	[M + Na]^+^	1.4%	1.9%	3.6%	2.5%	0.0%	0.0%	0.0%	5.4%
H_2_N	568.4	[M + Na]^+^	0.5%	0.3%	0.9%	1.8%	53.7%	50.1%	54.7%	1.1%
H_3_N	730.4	[M + Na]^+^	0.0%	0.5%	0.4%	0.5%	14.1%	13.9%	15.2%	1.3%
H_4_N	892.5	[M + Na]^+^	0.0%	0.0%	0.3%	0.3%	11.9%	13.5%	12.6%	1.4%
H_5_N	1054.5	[M + Na]^+^	0.0%	0.0%	0.2%	0.0%	3.2%	4.0%	3.2%	0.0%
H_6_N	1216.5	[M + Na]^+^	0.0%	0.0%	0.0%	0.0%	1.3%	1.5%	1.2%	0.0%
H_7_N	1378.5; 700.9	[M + Na]^+^; [M + 2Na]^2+^	0.0%	0.0%	0.0%	0.0%	0.6%	0.7%	0.5%	0.0%
H_8_N	1540.4; 782.0	[M + Na]^+^; [M + 2Na]^2+^	0.0%	0.0%	0.0%	0.0%	0.5%	0.5%	0.4%	0.0%
H_9_N	1702.8; 863.0	[M + Na]^+^; [M + 2Na]^2+^	0.0%	0.0%	0.0%	0.0%	0.2%	0.2%	0.3%	0.0%
HS	516.3	[M–H + 2Na]^+^	0.8%	0.0%	0.2%	0.4%	0.1%	0.1%	0.0%	1.0%
H_2_S	678.5; 656.5	[M–H + 2Na]^+^; [M + Na]^+^	2.7%	1.5%	2.3%	2.3%	0.7%	0.5%	0.6%	2.2%
NS			0.0%	0.0%	0.0%	0.0%	0.0%	0.0%	0.0%	0.0%
HNS	719.5; 697.6	[M–H + 2Na]^+^; [M + Na]^+^	2.9%	0.7%	1.6%	1.6%	0.4%	0.6%	0.5%	2.7%
N_2_S	760.3; 738.4	[M–H + 2Na]^+^; [M + Na]^+^	0.0%	0.0%	0.1%	0.2%	0.0%	0.0%	0.0%	0.0%
H_3_N_2_S	1246.8; 635.1	[M–H + 2Na]^+^; [M–H + 3Na]^2+^	0.0%	0.0%	0.0%	0.0%	0.0%	0.0%	0.0%	18.7%
H_5_N_3_S	898.3; 887.6	[M–H + 3Na]^2+^; [M + 2Na]^2+^	0.0%	0.2%	0.0%	0.0%	0.0%	0.0%	0.0%	3.2%
H_5_N_3_S_2_	1055.1; 1044.1	[M–2 H + 4Na]^2+^; [M–H + 3Na]^2+^	0.0%	0.0%	0.0%	0.0%	0.0%	0.0%	0.0%	8.7%
H_6_N_4_S_2_	1237.4	[M–2 H + 4Na]^2+^	0.0%	0.0%	0.0%	0.0%	0.0%	0.0%	0.0%	0.0%
H_6_N_4_S_3_			0.0%	0.0%	0.0%	0.0%	0.0%	0.0%	0.0%	0.0%
H_7_N_5_S_2_			0.0%	0.0%	0.0%	0.0%	0.0%	0.0%	0.0%	0.0%
H_7_N_5_S_3_			0.0%	0.0%	0.0%	0.0%	0.0%	0.0%	0.0%	0.0%
H_3_(SO_3_)N_2_S	1348.2; 674.5	[M–2 H + 3Na] ^+^; [M–H + 3Na]^2+^	0.0%	0.0%	0.0%	0.0%	0.0%	0.0%	0.0%	2.4%
H_5_(SO_3_)N_3_S	1854.3; 949.4	[M–H + 2Na]^+^; [M–2 H + 4Na]^2+^	0.0%	0.0%	0.0%	0.0%	0.0%	0.0%	0.0%	0.9%
H_5_(SO_3_)N_3_S_2_	2166.6; 1083.8	[M–2 H + 3Na] ^+^; [M–H + 3Na]^2+^	0.0%	0.0%	0.0%	0.0%	0.0%	0.0%	0.0%	1.0%
H_6_(SO_3_)N_4_S_3_			0.0%	0.0%	0.0%	0.0%	0.0%	0.0%	0.0%	0.0%
H_2_NS			0.0%	0.0%	0.0%	0.0%	0.0%	0.0%	0.0%	0.0%
S_2_			0.0%	0.0%	0.0%	0.0%	0.0%	0.0%	0.0%	0.0%
HX			0.0%	0.0%	0.0%	0.0%	0.0%	0.0%	0.0%	0.0%
H_2_X			0.0%	0.0%	0.0%	0.0%	0.0%	0.0%	0.0%	0.0%
HNX	606.5; 584.3	[M–H + 2Na]^+^; [M + Na]^+^	0.0%	0.5%	0.0%	0.0%	0.0%	0.0%	0.0%	0.0%
HNSX			0.0%	0.0%	0.0%	0.0%	0.0%	0.0%	0.0%	0.0%
SX			0.0%	0.0%	0.0%	0.0%	0.0%	0.0%	0.0%	0.0%
N_2_NeuGc			0.0%	0.0%	0.0%	0.0%	0.0%	0.0%	0.0%	0.0%
H_2_	365.2	[M + Na]^+^	48.7%	35.8%	46.7%	32.4%	7.1%	4.9%	3.2%	26.6%
H_3_	527.3	[M + Na]^+^	5.2%	3.8%	6.2%	5.6%	0.9%	2.9%	1.1%	6.5%
H_4_	689.5	[M + Na]^+^	1.6%	0.5%	2.2%	0.7%	0.4%	1.0%	0.8%	2.8%

*Comp* composition, *Fuc* Fucosidosis, *GM1-gang* G_M1_-gangliosidosis, *Sial* sialidosis

### Urinary glycans in α-mannosidosis

**Fig. 4:**
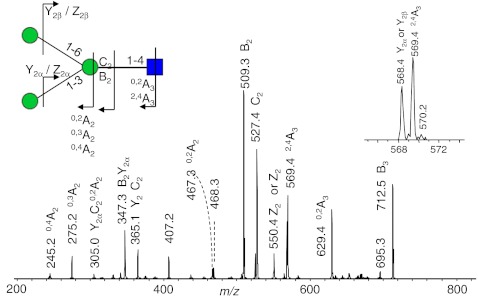
Positive-ion fragmentation mass spectrum of the monosodiated tetrasaccharide Man_3_GlcNAc_1_ (precursor ion at *m/z* 730.6) from urine of a α-mannosidosis patient. *Green circle* = mannose, *blue square* = *N*-acetylglucosamine

Three urine samples of three different α-mannosidosis patients from two different families were analyzed. In all three samples, 17 endo-β-*N*-acetylglucosaminidase cleavage products of mannose-rich oligosaccharides of composition Hex_2-9_HexNAc_1_ were detected [33, 45] (Table 2, Electronic supplementary material S2). The proposed structures are derived from literature [32, 33, 45] as well as from the obtained tandem MS data. An example of a fragment ion spectrum of the major Hex_3_HexNAc_1_ isomer is shown in Fig. 4. The ^0,2^A_3_ and ^2,4^A_3_ ions are typical for a 4-substituted HexNAc at the reducing end [41–43]. The cross-ring fragment ^0,3^A_2_ (*m/z* 275.2) is indicative for a 6-substituted hexose [41–43]. The B_2_Y_2α_ ion (D-ion, *m/z* 347.3) reveals the composition of the 6-antenna [42]. Histograms giving the relative abundances of the observed glycans are shown in Fig. 3b–d. The three urine samples resulted in very similar profiles including a prominent signal corresponding to Hex_2_HexNAc_1_. The whole set of oligomannosidic structures was detected (Hex_2–9_HexNAc_1_) showing decreasing signals with increasing size.

### Urinary glycans in G_M1_-gangliosidosis

**Fig. 5:**
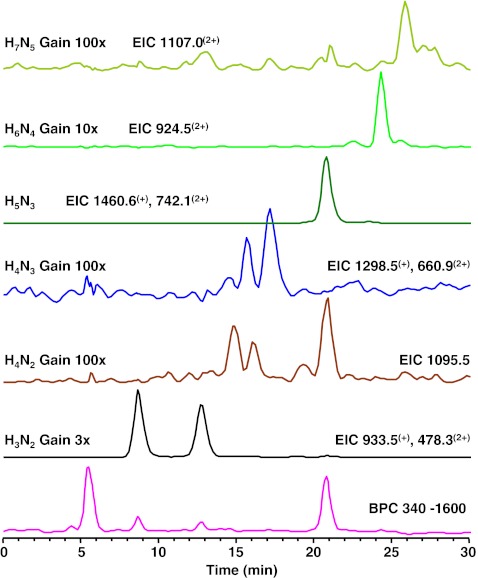
Separation of oligosaccharides in urine of a G_M1_-gangliosidosis patient. *H* hexose, *N N*-acetylhexosamine, *BPC* base peak chromatogram

**Fig. 6:**
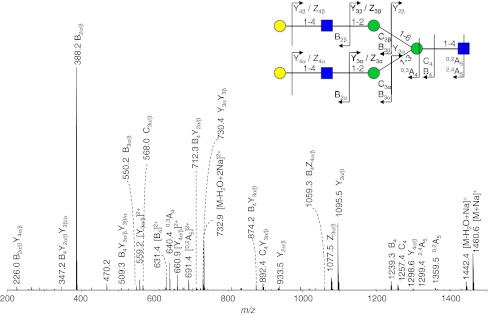
Positive-ion fragmentation mass spectrum of the disodiated diantennary oligosaccharide Hex_5_HexNAc_3_ (precursor ion at *m/z* 742.1) from urine of a G_M1_-gangliosidosis patient. *Yellow circle* galactose, *green circle* mannose, *blue square* *N*-acetylglucosamine

Extracted ion chromatograms of the disease-related glycans found in the urine of a G_M1_-gangliosidosis patient are represented in Fig. 5. Twenty glycan compositions were detected, and six of those structures with the composition Hex_3–7_HexNAc_2–5_ are presumably disease-related (Table 2, Electronic supplementary material Table S3). The compositions as well as the tandem mass spectrometric data (see Electronic supplementary material Table S3) suggest these glycans to be endo-β-*N*-acetylglucosaminidase cleavage products of complex type *N*-glycans. Composition Hex_3_HexNAc_2_ was interpreted as monoantennary and Hex_5_HexNAc_3_ as diantennary structure. Species carrying additional Hex_1_HexNAc_1_ units were found to be attached resulting in Hex_6_HexNAc_4_ and Hex_7_HexNAc_5_ species carrying more antennae as well as LacNAc repeats [9, 34, 35]. In addition, a relatively low amount of a trisaccharide with the composition Hex_1_HexNAc_1_HexonA_1_ was detected (Table 2). A histogram showing the relative abundance of the observed glycans is given in Fig. 3e with high signals corresponding to Hex_3_HexNAc_2_ and Hex_5_HexNAc_3_. The tandem MS spectrum of the disodiated diantennary *N*-glycan with the composition Hex_5_HexNAc_3_ (*m/z* 742.1) is shown in Fig. 6. The cross-ring fragments ^0,2^A_5_ and ^2,4^A_5_ are typical for a 4-substituted reducing end HexNAc [41–43]. The fragment ion B_4_Y_2α_ (D-ion, *m/z* 712.3) reveals the composition of the 6-antenna [42]. The characterization of the two well-separated isomers with the composition Hex_3_HexNAc_2_ has been reported earlier [25].

### Urinary glycans in G_M2_-gangliosidosis

**Fig. 7:**
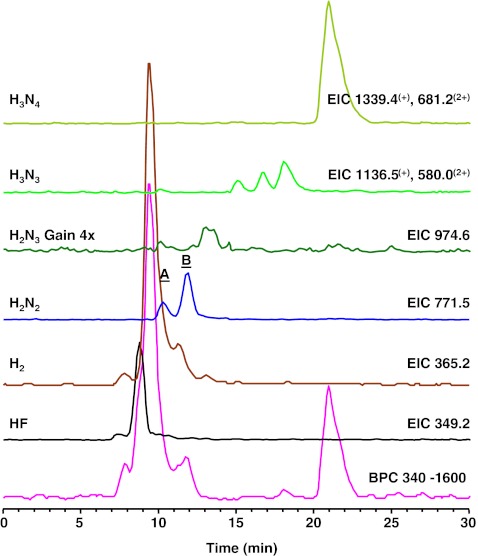
Separation of oligosaccharides in urine of a G_M2_-gangliosisis patient. Fragment ion spectra of the species A and B is shown in Fig. 8. *F* fucose, *H* hexose, *N N*-acetylhexosamine, *BPC* base peak chromatogram

**Fig. 8:**
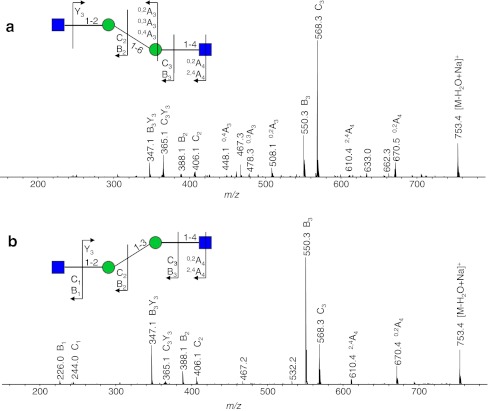
Positive-ion fragmentation mass spectra of two isomeric monosodiated tetrasaccharides Hex_2_HexNAc_2_ (precursor ion at *m/z* 771.5) from urine of a G_M2_-gangliosisis patient. The separation of A and B is shown in Fig. 7. *Green circle* mannose, *blue square* = *N*-acetylglucosamine

The analysis of the urine samples of two G_M2_-gangliosidosis patients revealed 11 G_M2_-gangliosidosis-related glycan isomers (Table 2, Electronic supplementary material Table S4) with the composition Hex_2–3_HexNAc_2–4_. The proposed structures reported in Electronic supplementary material Table S4 are based on our MS/MS results and on the known urinary oligosaccharides related to β-hexosaminidase deficiency in G_M2_-gangliosidos [15, 16]. Hex_2_HexNAc_2_ corresponds to a monoantennary, Hex_2_HexNAc_3_ to a bisected monoantennary, Hex_3_HexNAc_3_ to a diantennary, and Hex_3_HexNAc_4_ to a triantennary or bisected diantennary structure [16]. Figure 7 shows an excellent example of the isomeric separation of the two reported monoantennary glycans (Hex_2_HexNAc_2;_ *m/z* 771.5). The EIC corresponding to Hex_2_HexNAc_2_ (*m/z* 771.5) shows the separation of these isobaric structures (retention times 10.3 and 11.9 min; Fig. 7), and the MS/MS spectra are shown in Fig. 8. The observed series of B-ions are in accordance with a monosaccharide sequence of HexNAc-Hex-Hex-HexNAc for both isomers. Both oligosaccharides contain *N*-acetylhexosamine at the reducing end, which shows the cross-ring fragments ^0,2^A_4_ (*m/z* 670) and ^2,4^A_4_ (*m/z* 610) indicative for a 4-substituted *N*-acetylhexosamine [41–43]. The observed cross-ring fragments ^0,2^A_3_ (*m/z* 508), ^0,3^A_3_ (*m/z* 478), and ^0,4^A_3_ (*m/z* 448) observed for the adjacent hexose are typical for a 6-substitution. Based on the observed MS/MS data and literature data [15], glycan A was identified as the G_M2_-gangliosidosis urinary tetrasaccharide GlcNAc(β1-2)Man(α1-6)Man(β1-4)GlcNAc. A lack of A_3_ cross-ring fragments, which is typical for a 3-substituted sugar, indicates that glycan B is the isomer GlcNAc(β1-2)Man(α1-3)Man(β1-4)GlcNAc. Hence, the linkage-specific fragmentation allowed the assignment of the observed glycans to two urinary glycans related to G_M2_-gangliosidosis [15]. Moreover, the isomeric separation is emphasized by the different elution times of the three isomers corresponding to Hex_3_HexNAc_3_ registered in monosodidated (*m/z* 1136.5) as well as disodiated (*m/z* 580.0) form. Figure 3f and g show the relative abundance of the detected glycans for the two urine samples. In both samples, a high relative abundance of Hex_2_HexNAc_2_ (both samples 7.6%), Hex_2_HexNAc_3_ (1.3% and 1.2%), Hex_3_HexNAc_3_ (8.0% and 8.2%), and Hex_3_HexNAc_4_ (14.8% and 15.5%) was observed.

### Urinary glycans in sialidosis

Analysis of the urine of a sialidosis patient revealed eight disease-related sialylated oligosaccharides (Table 2, Electronic supplementary material S5). Structures with a high relative abundance such as Hex_3_HexNAc_2_ (6.5%), HexHexNAc (5.4%), Hex_3_HexNAc_2_Neu5Ac (18.7%), and Hex_5_HexNAc_3_Neu5Ac_2_ (8.7%) were detected (Fig. 3h). The presence of sulfated sialyloligosaccharides H_3–5_SO_3_N_2–3_ S_1–2_ is noteworthy [26].

## Discussion

Using a prototype capillary HPAEC-IPAD-MS system for analyzing a set of 54 glycans in eight urine samples from patients with lysosomal storage disorders such as fucosidosis, α-mannosidosis, G_M1_-gangliosidosis, G_M2_-gangliosidosis, and sialidosis (Table 1), we were able to find disease-related glycan structures. In addition, we identified glycan structures that are most probably diet- (human milk) or blood-group-related and are not related to the investigated disorders [37, 46–51] (see Table 2 and Electronic supplementary material Tables S1–S5). All urine samples, except for those of the mannosidosis patients, were found to contain a high relative amount of dihexose which is most likely a dietary product [37, 46, 47]. The presence of dietary products in urine is not surprising. We reported in a previous research about free oligosaccharides such as lactose, sialylhexose, and sialyllactose that we detected as major abundant carbohydrates in control urine samples of four healthy individuals [26].

Detection was performed using an ion trap mass spectrometer which was operated in automatic tandem MS mode resulting in informative fragment ion spectra for many glycans. Linkage-specific fragment ions [41–43] together with the known structural selectivity of high-performance anion-exchange chromatography [52–54] and literature knowledge on urinary oligosaccharides of LSDs [30, 55] made it possible to assign structures to most of the observed chromatographic signals.

Literature on fucosidosis reports that fucosylglycoasparagines are the most abundant glycoconjugates found in the urine of these patients [2, 56]. These glycoconjugates are not expected to show up in our analysis, as they will presumably adsorb to or pass through the membrane of the online desalter. This phenomenon is due to the high negative charge density of the fiber wall which is expected to result in strong interactions with cations such as glycopeptides entering the desalter [25]. Instead, we detected free fucosylsaccharides in the urine samples. The most abundant one is the disaccharide Fuc(α1-6)GlcNAc (Table 2, Electronic supplementary material Table S1, Figs. 2 and 3a) which is characteristic for this disorder [2, 56, 57]. Moreover, a trisaccharide with composition Hex_1_HexNAc_1_Fuc_1_ was found for fucosidosis (Fig. 3a). This trisaccharide is possibly the previously reported GalNAc(α1-3)[Fucα1-2)]Gal [56]. In addition, Tsay et al. [58] and Nishigaki et al. [59] reported the presence of a fucosylated decasaccharide, however, this structure has neither been detected by us nor by Strecker et al. [56].

Moreover, 17 endo-β-*N*-acetylglucosaminidase cleavage products including chromatographically separated isomers were detected in all three urine samples of patients suffering from α-mannosidosis (see Electronic supplementary material Table S2). These findings are in agreement with previous results reported by Matsuura et al. [33]. The authors identified in their study a similar number of endo-β-*N*-acetylglucosaminidase cleavage products [33]. However, while we observed three Hex_4_HexNAc_1_ isomers, three Hex_5_HexNAc_1_ isomers, and one Hex_7_HexNAc_1_ isomer, these authors found two, two, and three isomers, respectively (see Electronic supplementary material Table S2).

One of the three isomers of Hex_3_HexNAc_3_ found in the urine samples from patients suffering from G_M2_ gangliosidosis is in accordance with the diantennary structure with the core trimannose previously described by Strecker et al. [15, 60] while the other two isomers are probably monoantennary structures decorated with a GlcNAc(β1-3)Gal(β1-4)GlcNAc(β1-2) antenna. In the current study, we reported eight glycan structures that are related to sialidosis (see Electronic supplementary material Table S5). Of these eight glycans, five have been previously identified in sialidosis [39, 40, 50, 61]. We interpreted the structure of the glycan with the composition Hex_1_HexNAc_1_Neu5Ac_1_ as Neu5Ac(α2-3/6)Gal(β1-4)GlcNAc and being related to sialidosis [50], although, based on our data, we cannot exclude that Neu5Ac(α2-3/6)Gal(β1-4)GlcNAc might be sialyllactosamine from milk [37, 62, 63]. All sialidosis-relevant carbohydrate structures described here are terminated with sialic acid residues, in accordance with the primary defect in exo-α-sialidase. In addition, three *O*-sulfated oligosaccharides with terminal sialic acid residues were detected in the urine sample (Table 2) showing structures previously detected by us in galactosialidosis [26]. This may imply that the *O*-sulfated carbohydrates reported here are indeed related to the exo-α-sialidase deficiency found in both galactosialidosis and sialidosis. MS detection in the positive ion mode is known to be less sensitive for negatively charged glycans such as sialyl- and *O*-sulfated-oligosaccharides. Therefore, this sample has also been analyzed in the negative ion mode and indeed more sialylated-, *O*-sulfated-glycans, and glycans having reducing end aldohexonic acid residue were observed due to the improved sensitivity for the detection of negatively charged molecules (data not shown).

All together, this publication shows the value of capillary HPAEC-IPAD-MS for analyzing oligosaccharides in clinical urine samples without the need for derivatization. This prototype analytical system features femtomol sensitivity for both pulsed amperometric detection and mass spectrometric detection [25] allowing the relatively low abundant *O*-sulfated-glycan moieties to be detected. In contrast to other liquid chromatography methods relying on reducing end labeling for detection and/or separation [64, 65], HPAEC-IPAD as well as HPAEC-MS do not depend on glycan labeling. Consequently, we were able to detect an oxidized oligosaccharide with an innermost aldohexonic acid residue in the G_M1_-ganglioside urine sample. Moreover, the setup used in this study enables the separation of isomeric glycans. Based on the efficient fragment ion analysis using an ion trap instrument, informative fragment spectra of sodium adducts can be obtained with minute amounts of material, allowing insights into defects of glycoconjugates degradation, and investigation of metabolic and catabolic pathways.

Although the used instrumentation is a prototype, similar analyses can be performed using commercially available narrow bore ion chromatographs [66], with presumably less sensitivity due to the bigger dimensions and higher flow rates. The desalter in such a system is based on a flat semi-permeable cation exchange membrane and regenerated by electrolysis of water [67, 68].

LC-MS in general and the here described method in particular are analytically powerful. In the current research paper, we demonstrated that the HPAEC-MS technology in combination with MS/MS information on structural isomers is suitable for determining characteristic glycan fingerprints in lysosomal storage diseases which may have diagnostic potential.

## Electronic supplementary material

Below is the link to the electronic supplementary material.

ESM 1 (PDF 662 kb)
