# Supplementary material for: Analysis of urinary oligosaccharides in lysosomal storage disorders by capillary high-performance anion-exchange chromatography–mass spectrometry
Source: Anal Bioanal Chem. 2012 Apr 20;403(6):1671–83. doi: 10.1007/s00216-012-5968-9 (PMC3354319; doi:10.1007/s00216-012-5968-9)
Supplement: Supplementary file 1 — (PDF 662 kb) [file 216_2012_5968_MOESM1_ESM.pdf]

Analytical and Bioanalytical Chemistry

Electronic Supplementary Material

Analysis of urinary oligosaccharides in lysosomal storage disorders by capillary high-performance anion-exchange chromatography–mass spectrometry

Cees Bruggink, Ben J. H. M. Poorthuis, André M. Deelder, Manfred Wuhrer

Table S1. Oligosaccharide species detected in the fucosidosis sample U1. Comp., composition; Ret., retention; Rel. area., relative area.

	Comp.	Registered *m/z*	Charge state	Ret. time (min)	Fragment ions	Rel. area	Proposed structure
						U1	
**Disease related glycans**	NF	**390.2**	**[M+Na]$^{+}$**	**8.8**	244.0 N; 226.0 N-18; 187.2 F; 169.1 F-18	**10.6%**	**Fuc(α1-6)GlcNAc [2,29,30,37,44]**
	H_2NF	**714.5**	**[M+Na]$^{+}$□**	**9.1**		**0.3%**	
	H_3N_2F	**1079.4**	**[M+Na]□$^{+}$**	**9.2**		**1.3%**	
	HNSF	**865.4**	**[M-H+2Na]$^{+}$**	**22.7**	719.5 HNS; 552.4 HNF; 534.3 HNF-18; 516.2 HS; 498.0 HS-18; 405.9 HN; 388.1 HN-18; 336.0 S-18; 318.1 S-2x18	**0.8%**	**NeuAc(α2-3/6)Gal(β1-4)[Fuc(α1-3)]GlcNAc**
				**24.0**	552.2 HNF; 534.1 HNF-18; 516.0 HS; 498.2 HS-18; 354.2 S; 336.0 S-18	**1.6%**	
**Other glycans**	H_2NF_2	**860.5**	**[M+Na]$^{+}$**	**8.9**	714.4 H_2NF; 696.1 H_2NF-18; 657.3 H_2F_2; 568.3 H_2N; 552.4 HNF; 550.4 H_2N-18; 534.3 HNF-18; 532.5 H_2N-2x18; 405.9 HN; 388.1 HN-18; 370.3 HN-2x18; 349.1 HF; 244.0 N	**2.3%**	**GalNAc(α1-3)[Fuc(α1-2)]Gal(β1-4)[Fuc(α1-3)]Glc [37]**
	HF	**349.2**	**[M+Na]$^{+}$**	**8.9**		**2.6%**	**Fuc(α1-2)Gal [37]**
	H_2F	**511.3**	**[M+Na]$^{+}$**	**9.4**		**0.3%**	**Fuc[Gal(β1-4)Glc [37,46,47]**
	HNF	**552.5**	**[M+Na]$^{+}$**	**8.9**	406.1 HN; 388.1 HN-18; 372.1 NF-18; 244.0 N; 226.0 N-18; 208.0 N-2x18; 203.0 H; 187.1 F; 185.0 H-18; 169.0 F-18	**14.2%**	**GalNAc(α1-3)[Fuc(α1-2)]Gal [37]**
	H_2	**365.2**	**[M+Na]$^{+}$**	**9.7**	203.0 H; 185.0 H-18	**49.1%**	
	H_3	**527.3**	**[M+Na]$^{+}$**	**11.0**	365.1 H_2; 347.1 H_2-18; 203.1 H; 185.0 H-18	**2.4%**	
				**12.7**	365.1 H_2; 347.1 H_2-18; 203.1 H; 185.0 H-18	**1.5%**	
				**23.6**	365.1 H_2; 347.1 H_2-18; 203.0 H; 185.0 H-18	**1.4%**	
	H_4	**689.5**	**[M+Na]$^{+}$**	**23.9**	527.3 H_3; 509.2 H_3-18; 365.3 H_2; 346.9 H_2-18	**0.5%**	
				**25.3**	527.3 H_3; 509.2 H_3-18; 365.1 H_2; 347.0 H_2-18	**1.2%**	
	HN	**406.2**	**[M+Na]$^{+}$**	**9.4**		**1.4%**	
	H_2N	**568.4**	**[M+Na]$^{+}$**	**9.8**		**0.5%**	
	H_2N_2	**771.5**	**[M+Na]$^{+}$**	**14.3**		**1.6%**	

	HNS	**719.5/ 697.2**	**$[M+Na]^+$ $[M-H+2Na]^+$**	**25.2**	516.2 HS; 498.1 HS-18; 406.1 HN; 354.0 S; 336.0 S-18; 226.0 N	**3.0%**	**NeuAc(α2-3/6)Gal(β1-4)GlcNAc [50]**
	HS	**516.1**	**$[M-H+2Na]^+$**	**25.6**		**0.8%**	**Neu5Ac-Hex**
	H_2S	**678.5**	**$[M-H+2Na]^+$**	**25.7**	498.2 HS-18; 354.0 S; 365.1 H_2; 336.0 S-18	**2.7%**	**NeuAc(α2-3/6)Gal(β1-4)Glc [50]**

Table S2. Oligosaccharide species detected in the α-mannosidosis samples U5, U6, and U7. Comp., composition; Ret., retention.

	Comp.	Registered *m/z*	Charge state	Ret. time (min)	Fragment ions	Relative area			Proposed structure
						**U5**	**U6**	**U7**	
**Disease related glycans**	H_2N	**568.4**	**$[M+Na]^{+}$**	**5.8**	406.2 HN; 388.1 HN-18; 365.2 H_2; 347.2 H_2-18; 244.1 N; 226.0 N-18; 203.1 H	**52.9%**	**49.6%**	**53.7%**	**Man(α1-2)Man(α1-3)Man(β1-4)GlcNAc [31-33,45]**
	H_3N	**730.4**	**$[M+Na]^{+}$**	**8.1**	568.3 H_2N; 550.3 H_2N-18; 527.3 H_3; 509.2 H_3-18; 388.2 HN-18; 365.1 H_2; 347.2 H_2-18; 329.0 H_2-2x18; 244.1 N; 226.0 N-18	**1.2%**	**1.4%**	**1.0%**	**Man(α1-6)[Man(α1-3)]Man(β1-4)GlcNAc [33,45]**
				**8.9**	527.2 H_3; 509.2 H_3-18; 406.2 HN; 388.1 HN-18; 365.3 H_2; 347.2 H_2-18; 329.2 H_2-2x18; 244.2 N; 226.2 N-18	**12.7%**	**12.3%**	**13.9%**	**Man(α1-2)Man(α1-3)Man(β1-4)GlcNAc [31-33,45]**
	H_4N	**892.5**	**$[M+Na]^{+}$□**	**10.6**	730.2 H_3N; 712.3 H_3N-18; 689.4 H_4; 671.3 H_4-18; 527.1 H_3; 509.3 H_3-18; 406.3 HN; 347.1 H_2-18	**0.4%**	**0.4%**	**0.3%**	
				**12.8**	730.2 H_3N; 712.3 H_3-18; 689.3 H_4; 671.3 H_4-18; 568.4 H_2N; 550.3 H_2N-18; 527.2 H_3; 509.3 H_3-18; 491.3 H_3-2x18; 406.2 HN; 388.3 HN-18; 365.3 H_2; 347.2 H_2-18; 329.2 H_2-2x18; 244.2 N	**10.7%**	**12.5%**	**11.1%**	**Man(α1-2)Man(α1-2)Man(α1-3)Man(β1-4)GlcNAc [32,33,45]**
				**15.4**	730.2 H_3N; 712.3 H_3N-18; 689.3 H_4; 671.3 H_4-18; 568.2 H_2N; 527.3 H_3; 509.2 H_3-18; 491.1 H_3N-2x18; 406.0 HN; 388.1 HN-18; 365.1 H_2; 347.2 H_2-18	**0.6%**	**0.5%**	**0.9%**	**Man(α1-3)Man(α1-6)[Man(α1-3)]Man(β1-4)GlcNAc [32]**
	H_5N	**1054.5**	**$[M+Na]^{+}$□**	**15.9**	874.5 H_4N-18; 851.3 H_5; 833.4 H_5-18; 712.8 H_3N-18; 689.3 H_4; 671.3 H_4-18; 568.3 H_2N; 550.2 H_2N-18; 527.1 H_3; 509.3 H_3-18; 491.2 H_3-2x18	**0.7%**	**0.6%**	**0.7%**	**Man(α1-3)Man(α1-6)[Man(α1-2)Manα1-3)]Man(β1-4)GlcNAc [32,33,45]**
				**16.7**	851.4 H_5; 833.4 H_5-18; 712.3 H_3N-18; 689.4 H_4; 671.3 H_4-18; 653.4 H_4-2x18; 568.3 H_2N; 527.1 $H_{3'}$; 509.3 H_3-18; 365.1 $H_{2'}$; 347.1 H_2-18	**2.4%**	**3.4%**	**2.4%**	**Man(α1-6)[Man(α1-3)]Man(α1-6)[Man(α1-3)]Man(β1-4)GlcNAc [32,33,45]**
				**18.6**	892.3 H_4N; 851.3 H_5; 833.1 H_5-18; 730.3 H_3N; 689.3 H_4; 671.4 H_4-18; 568.4 H_2N; 550.0 H_2N-18; 509.2 H_3-18; 491.1 H_3-2x18; 365.1 $H_{2'}$; 346.9 H_2-18	**0.2%**	**0.1%**	**0.3%**	

	H_6N	**1216.5**	**$[M+Na]^+$**	**19.2**	1054.3 H_5N; 1036.4 H_5N-18; 1013.4 H_6; 995.4 H_6-18; 892.4 H_4N; 874.4 H_4N-18; 851.3 H_5; 833.3 H_5-18; 730.3 H_3N; 671.4 H_4-18; 653.3 H_4-2x18; 568.3 H_2N; 527.3 H_3; 509.3 H_3-18; 491.2 H_3-2x18	**0.5%**	**0.6%**	**0.4%**	**Man(α1-2)Man(α1-6)[Man(α1-3)]Man(α1-6)[Man(α1-3)]Man(β1-4)GlcNAc [32,33,45]**
				**19.9**	1054.5 H_5N; 1036.4 H_5N-18; 1013.3 H_6; 995.4 H_6-18; 892.3 H_4N; 851.3 H_5; 833.4 H_5-18; 730.2 H_3N; 689.3 H_4; 671.4 H_4-18; 527.5 H_3; 509.1 H_3-18; 347.1 H_2-18	**0.7%**	**0.9%**	**0.8%**	**Man(α1-6)[Man(α1-3)]Man(α1-6)[Man(α1-2)Man(α1-3)]Man(β1-4)GlcNAc [32,33,45]**
				**21.4**		**0.1%**	**0.1%**	**0.2%**	**Man(α1-2)Man(1-6)[Man(α1-3)]Man(α1-6)[Manα1-3)]Man(β1-4)GlcNAc [32,33,45]**
	H_7N	**700.9**<br>**1378.5**	**$[M+2Na]^{2+}$**<br>**$[M+Na]^+$**	**21.7**	1198.3 H_6N-18; 1175.4 H_7; 1157.3 H_7-18; 1054.5 H_5N; 1036.4 H_5N-18; 1013.2 H_6; 995.4 H_6-18; 892.3 H_4N; 874.3 H_4N-18; 851.2 H_5; 833.1 H_5-18; 712.4 H_3N; 689.4 H_4; 671.4 H_4-18; 509.1 H_3-18; 491.3 H_3-2x18	**0.6%**	**0.7%**	**0.5%**	**Man(α1-2){Man(α1-6)[Man(α1-3)]Man(α1-6)[Man(α1-2)Man(α1-3)]Man(β1-4)GlcNAc [32,33,45]**
	H_8N	**782.0**<br>**1540.4**	**$[M+2Na]^{2+}$**<br>**$[M+Na]^+$**	**15.8**		**0.1%**	**0.1%**		
				**23.9**	671.1 H_4-18; 346.9 H_2-18	**0.3%**	**0.4%**	**0.4%**	**2xMan(α1-2){Man(α1-6)[Man(α1-3)]Man(α1-6)[Man(α1-2)Man(α1-3)]Man(β1-4)GlcNAc [32,33,45]**
				**25.5**		**0.1%**	**0.1%**		
	H_9N	**863.0**<br>**1702.8**	**$[M+2Na]^{2+}$**<br>**$[M+Na]^+$**	**25.8**		**0.2%**	**0.2%**	**0.3%**	**Man(α1-2)Man(α1-6)[Man(α1-2)Man(α1-3)]Man(α1-6)[Man(α1-2)Man(α1-2)Man(α1-3)]Man(β1-4)GlcNAc [32,33,45]**
**Other glycans**	H_2	**365.2**	**$[M+Na]^+$**	**5.3**		**7.0%**	**4.8%**	**3.2%**	
	H_3	**527.3**	**$[M+Na]^+$**	**6.8**		**0.2%**	**0.1%**	**0.2%**	
				**7.8**	365.2 H_2; 347.2 H_2-18; 203.0 H	**0.3%**	**0.8%**	**0.5%**	
				**9.3**			**0.2%**		
				**9.6**	365.2 H_2; 347.2 H_2-18; 203.0 H; 185.0 H-18	**0.2%**	**0.5%**	**0.2%**	
				**15.7**		**0.1%**	**0.2%**		
				**16.8**		**0.0%**	**0.2%**		
				**17.5**	365.2 H_2; 347.3 H_2-18; 203.0 H; 185.1 H-18	**0.1%**	**0.5%**	**0.2%**	

				**18.7**			**0.3%**		
				**20.7**			**0.2%**		
	H_4	**689.4**	**$[M+Na]^+$**	**8.4**	527.2 H_3; 509.3 H_3-18; 365.2 H_2; 347.2 H_2-18		**0.4%**	**0.2%**	
				**25.7**	527.3 H_3; 509.2 H_3-18; 365.2 H_2; 347.2 H_2-18	**0.4%**	**0.6%**	**0.6%**	
	HN	**406.3**	**$[M+Na]^+$**	**4.3**		**1.4%**	**1.0%**	**1.6%**	
	H_2N_2	**771.1**	**$[M+Na]^+$**	**10.2**		**0.2%**	**0.1%**	**0.1%**	
	H_3N_2	**933.5**	**$[M+Na]^+$**	**8**		**0.1%**	**0.2%**		
	H_4N_2	**1095.5**	**$[M+Na]^+$**	**8**					
				**18.6**					
	H_5N_3	**741.9**	**$[M+2Na]^{2+}$**	**21.4**				**0.2%**	
	H_2NF_2	**860.5**	**$[M+Na]^+$**	**4.1**	714.3 H_2NF; 696.3 H_2NF-18; 657.4 H_2F_2; 568.3 H_2N; 552.2 HNF; 550.2 H_2N-18; 534.3 HNF-18; 511.3 H_2F; 406.3 HN; 388.2 HN-18; 349.2 HF; 331.1 HF-18; 244.1 N	**0.7%**	**3.1%**	**0.7%**	**GalNAc(α1-3)[Fuc(α1-2)]Gal(β1-4)[Fuc(α1-3)]Glc [37]**
	HF	**349.2**	**$[M+Na]^+$**	**3.9**		**1.9%**	**1.6%**	**2.1%**	
	H_2F	**511.2**	**$[M+Na]^+$**	**9.8**		**0.2%**		**0.3%**	
	NF	**390.2**	**$[M+Na]^+$**	**3.1**		**0.1%**			
	HNF	**552.4**	**$[M+Na]^+$**	**3.8**	406.2 HN; 388.1 HN-18; 349.3 HF; 243.8 N; 226.2 N-18; 203.1 H	**0.6%**	**0.2%**	**1.2%**	**GalNAc(α1-3)[Fuc(α1-2)]Gal [37]**
	HS	**516.2**	**$[M-H+2Na]^+$**	**23.8**		**0.1%**	**0.1%**		
	H_2NF	**714.4**	**$[M+Na]^+$**	**4.6**		**0.9%**	**0.2%**	**0.9%**	
	HNS	**697.4**<br>**719.4**	**$[M+Na]^+$**<br>**$[M-H+2Na]^+$**	**22.5**		**0.4%**	**0.6%**	**0.5%**	
	H_2S	**656.5**<br>**678.3**	**$[M+Na]^+$**<br>**$[M-H+2Na]^+$**	**25.0**	516,7 HS; 498,0/476,2 HS-18; 365.3 H_2; 347.0 H_2-18; 354,3/332.3 S; 336,2/314.1 S-18	**0.7%**	**0.5%**	**0.6%**	**Neu5Ac(α2-3)Gal(β1-4)Glc [50]**

Table S3. Oligosaccharide species detected in the G_{M1}-gangliosidosis sample U2. X, hexonic acid; GluconA, gluconic acid; Comp., composition; Ret., retention; Rel. area., relative area.

	**Comp.**	**Registered *m/z***	**Charge state**	**Ret. time (min)**	**Fragment ions**	**Rel. area**	**Proposed structure**
						**U2**	
**Disease related glycans**	H_3N_2	**933.5**<br>**478.3**	**[M+Na]$^+$**<br>**[M+2Na]$^{2+}$**	**8.8**	730.4 H_3N; 712.3 H_3N-18; 568.3 H_2N; 550.3 H_2N-18; 406.2 HN; 388.2 HN-18; 365.2 H_2; 347.2 H_2-18	**8.5%**	**Gal(β1-4)GlcNAc(β1-2)Man(α1-6)Man(β1-4)GlcNAc [9,25,34-36,44]**
		**933.5**<br>**478.3**	**[M+Na]$^+$**<br>**[M+2Na]$^{2+}$**	**12.8**	730.4 H_3N; 712.4 H_3N-18; 568.3 H_2N; 550.3 H_2N-18; 406.2 HN; 388.2 HN-18; 365.1 H_2; 347.2 H_2-18	**7.2%**	**Gal(β1-4)GlcNAc(β1-2)Man(α1-3)Man(β1-4)GlcNAc [9,25,34-36,44]**
	H_4N_2	**1095.5**	**[M+Na]$^+$**	**15.0**	933.3 H_3N_2; 915.1 H_4N_2-18; 892.2 H_4N; 874.3 H_4N-18; 753.4 H_2N_2-18; 730.3 H_3N; 712.1 H_3N-18; 568.2 H_2N; 550.3 H_2N-18; 532.2 H_2N-2x18; 527.3 H_3; 509.0 H_3-18; 406.4 HN; 388.2 HN-18; 365.1 H_2	**0.2%**	**Gal(β1-4)GlcNAc(β1-2)Man(α1-6)[Man(α1-3)]Man(β1-4)GlcNAc [9]**
		**1095.5**	**[M+Na]$^+$**	**16.1**	933.3 H_3N_2; 892.2 H_4N; 874.4 H_4N-18; 730.3 H_3N; 568.2 H_2N; 550.3 H_2N-18; 509.2 H_3-18; 388.1 HN-18	**0.1%**	**Man(α1-6)[Gal(β1-4)GlcNAc(β1-2)Man(α1-3)]Man(β1-4)GlcNAc Yamashita, 1981 49 /id}[44]**
		**1095.5**	**[M+Na]$^+$**	**21.0**		**0.3%**	
	H_4N_3	**1298.5**<br>**660.9**	**[M+Na]$^+$**<br>**[M+2Na]$^{2+}$**	**15.8**		**0.2%**	
		**1298.5**<br>**660.9**	**[M+Na]$^+$**<br>**[M+2Na]$^{2+}$**	**17.3**	1077.7/550.3 H_4N_2-18; 933.3 H3N2; 550.3 H_2N-18 or H_4N_2-9; 388.1 HN-18 or H_2N_2-9	**0.4%**	**Gal(β1-4)GlcNAc(β1-2)[Gal(β1-4)GlcNAc(β1-4)]Man(α1-3)Man(β1-4)GlcNAc [35]**
	H_5N_3	**1460.6/**<br>**742.1**	**[M+Na]$^+$**<br>**[M+2Na]$^{2+}$**	**20.9**	1298.6 H_4N_3; 1280.5 H_4N_3-18; 1257.6 H_5N_2; 1239.6 H_5N_2-18; 1095.6 H_4N_2; 1077.6 H_4N_2-18; 1059.6 H_4N_2-2x18; 933.5 H_3N_2; 915.4 H_3N_2-18; 892.5 H_4N; 874.6 H_4N-18;771.4 H_2N_2; 730.5 H_3N; 712.4 H_3N-18; 694.3 H_3N-2x18; 568.4 H_2N; 550.3 H_2N-18; 532.4 H_2N-2x18; 527.3 H_3; 514.1 H_2N-3x18; 509.3 H_3-18; 405.9 HN; 388.2 HN-18; 370.2 HN-2x18; 365.3 H_2; 347.2 H_2-18; 329.2 H_2-2x18; 244.0 N; 226.0 N-18; 208.1 N-2x18	**26.2%**	**Gal(β1-4)GlcNAc(β1-2)Man(α1-6)[Gal(β1-4)GlcNAc(β1-2)Man(α1-3)]Man(β1-4)GlcNAc [9,34-36,44]**
	H_6N_4	**924.5**	**[M+2Na]$^{2+}$**	**22.7**	1442.7 H_5N_3-18; 1257.4 H_5N_2; 1239.5 H_5N_2-18; 892.3 H_4N; 651.7 H_4N_3-9; 570.8 H_3N_3-9; 388.2 HN-18	**0.2%**	

		**924.5**	**$[M+2Na]^+$**	**24.4**	1622.4 H_6N_3; 1501.4 H_4N_4; 1460.5 H_5N_3; 1442.5 H_5N_3-18; 1424.4 H_5N_3-2x18; 1303.6 H_3N_4-2x18; 1298.6 H_4N_3; 1280.6 H_4N_3-18; 1257.6 H_5N_2; 1239.5 H_5N_2-18; 1118.5 H_3N_3-18; 1095.4 H_4N_2; 1077.6 H_4N_2-18; 1059.3 H_4N_2-2x18; 915.4 H_3N_2-18; 892.2 H_4N; 874.4 H_4N-18; 843.4 H_5N_4; 838.2 H_4N-3x18; 822.9 H_6N_3; 813.7 H_6N_3-9; 762.4 H_4N_4; 753.2 H_2N_2-18, H_4N_4-9; 741.9 H_5N_3; 735.2 H_2N_2-2x18; 732.9 H_5N_3-9; 730.2 H_3N; 712.3 H_3N-18; 660.8 H_4N_3; 640.3 H_5N_2; 570.3 H_3N_3; 568.3 H_2N; 559.3 H_4N_2; 552.6 H_3N_3-3x9;550.2 H_2N-18; 406.2 HN; 388.2 HN-18; 347.2 H_2; 329.2 H_2-18	**2.5%**	**Gal(β1-4)GlcNAc(β1-2)Man(α1-6)[Gal(β1-4)GlcNAc(β1-2)][Gal(β1-4)GlcNAc(β1-4)]Man(α1-3)]Man(β1-4)GlcNAc [9,34-36]**
		**924.5**	**$[M+2Na]^+$**	**25.7**	1604.5 H_6N_3-18; 1442.4 H_5N_3-18; 1257.4 H_5N_2; 1239.2 H_5N_2-18; 1095.4 H_4N_2; 1077.5 H_4N_2-18; 892.5 H_4N 874.4 H_4N-18; 822.8 H_6N_3; 753.3 H_2N_2-18; 730.3 H_3N; 550.2 H_4N_2; 388.2 HN-18	**0.2%**	**Gal(β1-4)GlcNAc(β1-3)Gal(β1-4)GlcNAc(β1-2)Man(α1-6)[Gal(β1-4)GlcNAc(β1-2)Man(α1-3)]Man(β1-4)GlcNAc [9,34-36]**
	H_7N_5	**1107.0**	**$[M+2Na]^{2+}$**	**25.9**	1825.7 H_6N_3; 1807.6 H_6N_3-18; 1663.6/843.4 H_5N_4; 1627.5 H_5N_4-2x18; 1622.6/822.7 H_6N_3; 1501.9 H_4N_4; 1460.6/741.7 H_5N_3; 1442.5 H_5N_3-18; 1298.5 H_4N_3; 1257.7 H_5N_2; 1239.5/631.4 H_5N_2-18; 1095.5 H_4N_2; 1077.4 H_4N_2-18; 1059.3 H_4N_2-2x18; 1016.9 H_6N_5-9; 1005.3 H_7N_4; 933.4/478.1 H_3N_2; 924.3 H_6N_4; 915.4 H_6N_4-9; 813.8 H_6N_3-9; 804.8 H_6N_3-2x9; 771.1 H_2N_2; 753.3 H_2N_2-18; 550.4 H_2N-18; 388.2 HN-18	**0.2%**	**Gal(β1-4)GlcNAc(β1-6)][Gal(β1-4)GlcNAc(β1-2)]Man(α1-6)[[Gal(β1-4)GlcNAc(β1-4)][Gal(β1-4)GlcNAc(β1-2)]Man(α1-3)]Man(β1-4)GlcNAc [9,34,36]**
**Other glycans**	H_2	**365.1**	**$[M+Na]^+$**	**3.0**	203.0 H	**1.4%**	
				**4.6**	203.0 H	**0.1%**	
				**5.6**	203.0 H; 185.0 H-18	**30.4%**	
				**7.1**	203.1 H; 185.1 H-18	**0.5%**	
				**9.0**	203.1 H; 185.0 H-18	**2.3%**	
	HN	**406.3**	**$[M+Na]^+$**	**5.3**	226.1 N; 203.0 H; 185.0 H-18	**1.8%**	
	H_3	**527.3**	**$[M+Na]^+$**	**3.8**	365.1 H_2, 3471,1 H_2-18; 202.9 H	**0.2%**	
				**6.5**	365.2 H_2; 347.1 H_2-18; 203.0 H; 185.0 H-18	**1.1%**	
				**7.5**	365.1 H_2; 347.2 H_2-18; 203.1 H	**0.2%**	
				**14.0**	365.2 H_2; 347.2 H_2-18; 185.0 H-18	**0.3%**	
				**15.4**	365.1 H_2; 347.2 H_2-18; 203.0 H; 184.8 H-18	**1.3%**	
				**16.5**	365.2 H_2; 347.1 H_2-18; 202.9 H; 185.0 H-18	**0.3%**	

				19.2	365.2 H_2; 347.0 H_2-18; 203.0 H	0.2%	
	H_4	689.4	$[M+Na]^{+}$	22.1	527.1 H_3; 509.2 H_3-18; 347.2 H_2-18; 203.0 H	0.1%	
					527.2 H_3; 509.2 H_3-18; 365.3 H_2; 347.2 H_2-18; 203.1 H	0.4%	
	HF	349.2	[M+Na]□	2.9		0.2%	
				4.6		0.4%	
	H_2F	511.3	$[M+Na]^{+}$	4.6	365.2 H_2, 347.2 H_2-18; 203.1 H; 185.1 H-18	4.2%	Fuc{Gal(β1-4)Glc [37,46,47,56]
	H_2N	568.3	$[M+Na]^{+}$	6.2		0.3%	
	H_3N	730.4	$[M+Na]^{+}$	9.0	550.2 H_2N; 406.2 HN; 388.2 HN-18	0.5%	
				14.8	568.2 H_2N; 550.3 H_2N-18; 406.2 HN; 388.2 HN-18; 203.1 H	3.2%	
	H_2NF	714.4	$[M+Na]^{+}$	3.8	568.3 H_2N; 550.4 H_2N-18; 406.2 HN; 388.2 HN-18; 226.1 N; 203.0 H	0.9%	
	HNF	552.3	$[M+Na]^{+}$	3.8	406.2 HN; 388.2 HN-18; 203.1 H	0.6%	GalNAc(α1-3)[Fuc(α1-2)]Gal [37]
	H_2S	656.4<br>678.4	$[M+Na]^{+}$<br>$[M–H+2Na]^{+}$	25.3	476.1 HS-18; 365.3 H_2; 347.2 H_2-18; 314.3 S-18; 202.9 H	1.4%	NeuAc(α2-3)Gal(β1-4)Glc [37,50]
	HNS	697.4<br>719.4	$[M+Na]^{+}$<br>$[M-H+2Na]^{+}$	23.5	516.2 HS; 336.1 S-18	0.2%	NeuAc(α2-6)Gal(β1-4)GlcNAc [37,50]
			$[M+Na]^{+}$<br>$[M–H+2Na]^{+}$	25.0	539.1 NS-18; 406.5 HN; 226.0 N	0.4%	
	H_5N_3S	887.8	$[M+2Na]^{2+}$	20.7		0.2%	
	HNX	584.3/<br>606.5	$[M+Na]^{+}$<br>$[M–H+2Na]^{+}$	20.6		0.4%	

Table S4. Oligosaccharide species detected in the G_{M2}-gangliosidosis samples U3 and U4. Comp., composition; Ret., retention.

	Comp.	Registered *m/z*	Charge state	Ret. time (min)	Fragment ions	Relative Area		Proposed structure
						U3	U4	
Disease related glycans	H_2N_2	**771.5**	**$[M+Na]^+$**	**10.3**	568.3 H_2N; 550.3 H_2N-18; 406.1 HN; 388.1 HN-18; 365.1 H_2; 347.1 H_2-18		**1.6%**	**GlcNAc(β1-2)Man(α1-3)Man(β1-4)GlcNAc [15,16,44]**
		**771.5**	**$[M+Na]^+$**	**11.9**	568.3 H_2N; 550.3 H_2N-18; 406.1 HN; 388.1 HN-18; 365.1 H_2; 347.1 H_2N-18; 244.0 N; 226.0 N-18	**7.6%**	**6.0%**	**GlcNAc(β1-2)Man(α1-6)Man(β1-4)GlcNAc [15]**
	H_3N_3	**1136.5**<br>**580.0**	**$[M+Na]^+$**<br>**$[M+2Na]^{2+}$**	**15.1**	933.4 H_3N_2; 915.3 H_3N_2-18; 771.5 H_2N_2; 730.4 H_3N; 712.3 H_3N-18; 568.2 H_2N; 550.2 H_2N-18	**0.6%**	**1.1%**	**GlcNAc(β1-2)Man(α1-6)[GlcNAc(β1-2)Man(α1-3)]Man(β1-4)GlcNAc [15,16,44]**
		**1136.5**<br>**580.0**	**$[M+Na]^+$**<br>**$[M+2Na]^{2+}$**	**16.8**	933.4 H_3N_2; 915.3 H_3N_2-18; 730.5 H_3N; 712.4 H_3N-18; 550.3 H_2N-18	**3.6%**	**1.3%**	
		**1136.5**<br>**580.0**	**$[M+Na]^+$**<br>**$[M+2Na]^{2+}$**	**18.1**	933.5 H_3N_2; 915.3 H_3N_2-18; 730.5 H_3N; 712.3 H_3N-18; 568.3 H_2N; 550.2 H_2N-18; 347.1 H_2-18	**3.8%**	**5.8%**	
	H_3N_4	**681.2**	**$[M+2Na]^{2+}$**	**10.0**		**0.4%**		
		**1339.4**<br>**681.2**	**$[M+Na]^+$**<br>**$[M+2Na]^{2+}$**	**12.9**	1136.4 H_3N_3; 1118.5 H_3N_3-18; 974.6 H_2N_3; 933.3 H_3N_2; 915.4 H_3N_2-18; 730.6 H_3N; 712.3 H_3N-18; 568.4 H_2N; 550.4 H_2N-18; 532.3 H_2N-2x18; 406.1 HN; 387.9 HN-18	**5.5%**		**GlcNAc(β1-2)Man(α1-6)[GlcNAc(β1-2)[GlcNAc(β1-4)]Man(α1-3)]Man(β1-4)GlcNAc [44,44,60]**
		**681.2**	**$[M+2Na]^{2+}$**	**14.0**	1136.4/579.8 H_3N_3; 1118.5/570.9 H_3N_3-18; 974.3 H_2N_3; 933.4/478.2 H_3N_2; 915.3 H_3N_2-18; 897.4 H_3N_2-2x18; 771.3 H_2N_2; 753.4 H_2N_2-18; 712.2 H_3N-18; 591.4 HN_2-18; 568.4 H_2N; 388.2 HN-18; 244.0 N; 226.0 N-18	**7.1%**	**15.5%**	**GlcNAc(β1-2)[GlcNAc(β1-4)]Man(α1-6)[GlcNAc(β1-2)Man(α1-3)]Man(β1-4)GlcNAc [15]**
		**681.2**	**$[M+2Na]^{2+}$**	**17.5**	1136.4/579.8 H_3N_3; 1118.4/570.8 H_3N_3-18; 974.3 H_2N_3; 933.3/478.2 H_3N_2; 915.3/469.2 H_3N_2-18; 771.5 H_2N_2; 730.4 H_3N; 591.3 HN_2-18; 489.7 H_2N_3-9; 388.1 HN-18; 364.8 H_2; 347.1 H_2-18; 226.0 N-18	**1.7%**		
		**1339.4**<br>**681.2**	**$[M+Na]^+$**<br>**$[M+2Na]^{2+}$**	**20.0**	1136.4 H_3N_3; 1118.5 H_3N_3-18; 974.3 H_2N_3; 933.4 H_3N_2; 915.3 H_3N_2-18; 897.4 H_3N_2-2x18; 771.3 H_2N_2; 753.4 H_2N_2-18; 712.2 H_3N-18; 591.4 HN_2-18; 568.4 H_2N; 388.2 HN-18; 244.0 N; 226.0 N-18	**0.1%**		

	H_2N_3	**974.6**	**[M+Na]$^+$**	**1.1**	771.4 H_2N_2; 753.4 H_2N_2-18; 609.4 HN_2; 568.3 H_2N; 550.3 H_2N-18; 365.0 H_2; 347.1 H_2-18	**1.3%**	**1.2%**	**GlcNAc(β1-2)Man(α1-3)[GlcNAc(β1-4)]Man(β1-4)GlcNAc [16,44]**
**Other glycans**	H_2	**365.2**	**[M+Na]$^+$**	**7.8**	203.0 H		**0.3%**	
				**9.4**	203.0 H; 185.0 H-18	**47.0%**	**30.3%**	
				**11.3**	203.0 H; 185.0 H-18		**0.8%**	
	HN	**406.2**	**[M+Na]$^+$**	**8.8**	244.0 N; 226.0 N-18; 203.0 H; 185.2 H-18	**3.6%**	**2.5%**	
	H_3	**527.3**	**[M+Na]$^+$**	**7.8**			**0.2%**	
				**11.1**	365.1 H_2; 347.0 H_2-18; 185.2 H-18	**1.6%**	**0.8%**	
				**12.1**	365.0 H_2; 347.0 H_2-18; 203.1 H; 184.9 H-18	**0.7%**	**1.3%**	
				**14.2**		**0.8%**	**0.5%**	
				**16.2**	365.1 H_2; 347.0 H_2-18; 203.0 H; 185.1 H-18	**0.4%**	**1.4%**	
				**18.7**	365.1 H_2; 203.0 H; 185.0 H-18	**0.7%**	**0.7%**	
				**23.1**	365.1 H_2; 347.0 H_2-18; 202.9 H; 185.0 H-18	**2.0%**	**0.7%**	
	H_4	**689.5**	**[M+Na]$^+$**	**22.7**	527.2 H_3; 509.2 H_3-18; 365.0 H_2; 347.0 H_2-18; 203.0 H	**0.7%**	**0.3%**	
				**23.2**	527.2 H_3; 509.3 H_3-18; 365.0 H_2; 347.0 H_2-18	**1.5%**	**0.4%**	
	HF	**349.2**	**[M+Na]$^+$**	**8.8**	203.0 H; 185.1 H-18; 169.1 F-18	**0.7%**	**15.5%**	
	H_2F	**511.3**	**[M+Na]$^+$**	**9.8**	365.1 H_2; 347.1 H_2-18; 330.8 HF-18	**0.6%**	**0.7%**	
	NF	**390.2**	**[M+Na]$^+$**	**8.3**		**0.2%**		
	H_2N	**568.4**	**[M+Na]$^+$**	**9.7**	347.1 H_2-18	**0.9%**	**1.7%**	
				**11.9**		**0.1%**		
	H_3N	**730.4**	**[M+Na]$^+$**	**9.3**		**0.4%**	**0.5%**	
	H_4N	**892.4**	**[M+Na]$^+$**	**9.8**		**0.3%**	**0.3%**	
	H_5N	**1054.5**	**[M+Na]$^+$**	**11.0**		**0.2%**		
	H_2NF	**714.5**	**[M+Na]$^+$**	**9.4**	568.4 H_2N; 552.4 HNF; 550.3 H_2N-18; 534.3 HNF-18; 406.2 HN; 389.9 NF; 388.1 HN-18; 372.1 NF-18; 365.1 H_2; 244.1 N; 203.0 H	**0.6%**	**0.8%**	
	H_2NF_2	**860.4**	**[M+Na]$^+$**	**7.6**		**0.2%**	**0.1%**	

	HNF	**552.5**	**$[M+Na]^+$**	**8.8**	406.1 HN; 388.1 HN-18; 226.0 N-18; 207.8 N-2x18; 203.0 H; 185.1 H-18	**0.5%**	**0.2%**	
	H_2S	**656.5**<br>**678.5**	**$[M+Na]^+$**<br>**$[M–H+2Na]^+$**	**23.8**	498.2 HS-18; 364.8 H_2; 336.0 S-18	**2.3%**	**2.2%**	
	N_2S	**738.4**<br>**760.3**	**$[M+Na]^+$**<br>**$[M–H+2Na]^+$**	**25.1**		**0.1%**	**0.1%**	
	HNS	**719.4**	**$[M–H+2Na]^+$**	**23.5**	516.2 HS; 498.3 HS-18; 406.0 HN; 388.1 HN-18; 353.9 S; 336.0 S-18	**1.6%**	**1.5%**	**NeuAc(α2-6)Gal(β1-4)GlcNAc [37,50]**
	HNSF	**865.4**	**$[M–H+2Na]^+$**	**22.3**		**0.2%**		
		**865.4**	**$[M–H+2Na]^+$**	**24.3**		**0.1%**		
	HS	**516.4**	**$[M–H+2Na]^+$**	**23.8**	353.7 S; 335.9 S-18	**0.2%**	**0.4%**	
	HX	**381.2**<br>**403.2**	**$[M+Na]^+$**<br>**$[M–H+2Na]^+$**	**20.4**	240.9 X; 203.0 H		**3.6%**	**Gal(β1-4)GluconA [26]**

Table S5. Oligosaccharide species detected in the sialidosis samples. Comp., composition; Ret., retention; Rel. area., relative area.

	Comp.	Registered *m/z*	Charge state	Ret. time (min)	Fragment ions	Rel. area	Proposed structure
						**U8**	
**Disease related glycans**	HNS	**697.6**<br>**719.5**	**$[M+Na]^+$**<br>**$[M–H+2Na]^+$**	**21.3**	516.2 HS; 498.2 HS-18; 406.1 HN; 388.1 HN-18; 354.0 S; 336.1 S-18; 318.0 S-2x18; 226.0 N-18	**2.7%**	**NeuAc(α2-6)Gal(β1-4)GlcNAc [37,50]**
	H_3N_2S	**1224.5**<br>**1246.8**<br>**624.1**<br>**635.1**	**$[M+Na]^+$**<br>**$[M–H+2Na]^{\square+}$**<br>**$[M+2Na]^{2+}$**<br>**$[M–H+3Na]^{2+}$**	**21.8**	1043.4/533.3 H_3NS; 1025.4/524.3 H_3NS-18; 933.4/477.9 H_3N_2; 915.4 H_3N_2-18; 881.5/452.3 H_2NS; 863.3/443.0 H_2NS-18; 771.4 H_2N_2; 753.5 H_2N_2-18; 730.4 H_3N; 712.5 H_3N-18; 719.4 HNS; 701.4 HNS-18; 568.4 H_2N; 550.2 H_2N-18; 516.3 HS; 498.3 HS-18; 406.1 HN; 388.1 HN-18; 365.0 H_2; 347.3 H_2-18; 354.0 S; 336.0 S-18; 243.9 N; 226.0 N-18; 208.0 N-2x18; 203.1 H; 185.0 H-18	**18.7%**	**Neu5Ac(α2□3)Gal(β1□4)GlcNAc(β1□2)Man(α1□3)Man(β1□4)GlcNAc [38-40]**
	H_5N_3S	**887.6**<br>**898.3**	**$[M+2Na]^{2+}$**<br>**$[M–H+3Na]^{2+}$**	**23.7**	1460.5 H_5N_3; 1442.6 H_5N_3-18; 1408.6 H_4N_2S; 1390.3 H_4N_2S-18; 1257.6 H_5N_2; 1095.5 H_4N_2; 1077.6 H_4N_2-18; 1025.2 H_3NS-18; 915.4 H_3N_2-18; 892.2 H_4N; 881.3 H_2NS; 797.4 H_4N_3S-9; 796.9 H_5N_2S; 771.5 H_2N_2; 694.5 H_3N-2x18; 640.5 H_5N_2; 605.0 H_4NS-9; 568.2 H_2N; 541.0 H_4N_2-2x9; 388.2 HN-18; 336.1/314.0 S-18	**3.2%**	**Neu5Ac(α2-3/6)Gal(β1-4)GlcNAc(β1-2)Man(α1-6)[Gal(β1-4)GlcNAc(β1-2)Man(α1-3)]Man(β1-4)GlcNAc [38,39]**
	$H_5N_3S_2$	**1033.1**<br>**1044.1**<br>**1055.1**	**$[M+2Na]^{2+}$**<br>**$[M–H+3Na]^{2+}$**<br>**$[M–2H+4Na]^{2+}$**	**29.1**	1773.4/898.5 H_5N_3S; 1733.1/1755.3/889.4 H_5N_3S-18; 1571.4/797.5 H_4N_3S-18; 1570.5 H_5N_2S; 1460.2/741.9 H_5N_3; 1408.4 H_4N_2S; 1368.8 H_4N_2S-18; 1095.5 H_4N_2; 953.4 $H_5N_2S_2$; 915.5 H_3N_2-18; 892.1 H_4N; 701.2 HNS-18; 694.4 H_3N-2x18; 605.3 H_4NS-9; 596.1 H_4NS-2x9; 313.9 S	**8.7%**	**Neu5Ac(α2-3/6)Gal(β1-4)GlcNAc(β1-2)Man(α1-6)[Neu5Ac(α2-3/6)Gal(β1-4)GlcNAc(β1-2)Man(α1-3)]Man(β1-4)GlcNAc [38-40]**
	$H_6N_4S_2$	**1237.4**	**$[M–2H+4Na]^{2+}$**	**28.8**		**0.0%**	
	$H_3SO_3N_2S$	**1304.3**<br>**1348.2**<br>**664.0**<br>**674.5**<br>**685.8**	**$[M+Na]^+$**<br>**$[M–2H+3Na]^+$**<br>**$[M+2Na]^{2+}$**<br>**$[M-H+3Na]^{2+}$**<br>**$[M-2H+4Na]^{2+}$**	**21.8**	933.5 $H_3(SO_3)N_2$; 701.4 $H(SO_3)NS$-18; 645.6/634.9 H_3N_2S; 634.5 $H_2(SO_3)N$; 516.3 $H(SO_3)S$; 196.0 HN	**2.4%**	**Neu5Ac(α2-3)Gal(6SO3)(β1-4)GlcNAc(β1-2)Man(α1-3/6)Man(β1-4)GlcNAc [26]**
	$H_5SO_3N_3S$	**1854.3**<br>**1876.3**<br>**927.2**	**$[M-H+2Na]^+$**<br>**$[M-2H+3Na]^+$**<br>**$[M+2Na]^{2+}$**	**22.0**		**0.9%**	**Neu5Ac, SO3(6){Gal(β1-4)GlcNAc(β1-2)Man(α1-6)[Gal(β1-4)GlcNAc(β1-2)Man(α1-3)]Man(β1-4)GlcNAc [26]**

		**938.3**<br>**949.4**	**[M-H+3Na]$^{2+}$**<br>**M-2H+4Na]$^{2+}$**				
	$H_5SO_3N_3S_2$	**2166.6**<br>**1083.8**	**[M-2H+3Na]$^{+}$**<br>**[M–H+3Na]$^{2+}$**	**33.8**		**1.0%**	**2xNeu5Ac, SO3(6){Gal(β1-4)GlcNAc(β1-2)Man(α1-6)[Gal(β1-4)GlcNAc(β1-2)Man(α1-3)]Man(β1-4)GlcNAc [26]**
**Other glycans**	H_2	**365.2**	**[M+Na]$^{+}$**	**6.2**	203.0 H	**1.5%**	
				**7.4**	203.0 H;185.1 H-18	**18.9%**	
				**8.7**	202.9 H;185.1 H-18	**3.5%**	
				**10.2**	203.0 H;185.0 H-18	**2.4%**	
				**13.5**	203.0 H;185.1 H-18	**0.3%**	
	HS	**516.3**	**[M–H+2Na]$^{+}$**	**21.7**		**1.0%**	
	H_3	**527.3**	**[M+Na]$^{+}$**	**8.9**	365.2 H_2; 347.1 H_2-18; 203.1 H; 185.0 H-18	**3.2%**	
				**13.5**	365.1 H_2; 347.0 H_2-18; 203.1 H; 185.1 H-18	**0.3%**	
				**18.2**	365.1 H_2; 347.0 H_2-18; 203.1 H; 185.1 H-18	**0.6%**	
				**20.8**		**0.6%**	
				**22.5**	365.1 H_2; 347.1 H_2-18; 203.1 H; 185.0 H-18	**0.9%**	
				**28.1**		**0.7%**	
	H_4	**689.5**	**[M+Na]$^{+}$**	**19.4**		**0.0%**	
				**21.2**	527.3 H_3; 509.3 H_3-18; 365.0 H_2; 347.0 H_2-18; 203.0 H	**2.8%**	
	HN	**406.2**	**[M+Na]$^{+}$**	**7.4**	244.0 N; 226.0 N-18; 203.0 H; 185.1 H-18	**5.4%**	
	H_2N	**568.5**	**[M+Na]$^{+}$**	**7.5**	365 H_2; 347.0 H_2-18; 244.2 N	**1.1%**	
	H_3N	**730.6**	**[M+Na]$^{+}$**	**8.5**	568.4 H_2N; 550.2 H_2N-18; 527.4 H_3; 509.3 H_3-18; 406.0 HN; 388.2 HN-18; 364.9 H_2; 347.0 H_2-18	**1.3%**	
				**12.1**	568.3 H_2N; 550.3 H_2N-18; 509.3 H_3-18; 406.1 HN; 388.1 HN-18; 365.1 H_2; 347.2 H_2-18; 244.1 N; 226.0 N-18; 203.0 H		
	H_4N	**892.6**	**[M+Na]$^{+}$**	**10.3**	730.4 H_3N; 689.4 H_4; 671.2 H_4-18; 568.3 H_2N; 550.3 H_2N-18; 509.4 H_3-18; 406.0 HN; 347.1 H_2-18	**1.4%**	

	H_2N_2	**771.3**	**$[M+Na]^+$**	**9.9**	388.1 HN-18	**0.3%**	
	H_3N_2	**933.5**<br>**478.4**	**$[M+Na]^+$**<br>**$[M+2Na]^{2+}$**	**11.1**	771.5 H_2N_2; 753.5 H_2N_2-18; 730.5 H_3N; 712.4 H_3N-18; 609.4 HN_2; 568.4 H_2N; 550.3 H_2N-18; 527.3 H_3; 509.2 H_3-18; 406.2 HN; 388.2 HN-18; 365.1 H_2; 347.2 H_2-18	**2.1%**	
		**933.5**<br>**478.4**	**$[M+Na]^+$**<br>**$[M+2Na]^{2+}$**	**13.4**	771.4 H_2N_2; 753.5 H_2N_2-18; 730.4 H_3N; 712.4 H_3N-18; 568.4 H_2N; 550.4 H_2N-18; 509.4.3 H_3-18; 406.1 HN; 388.2 HN-18; 365.1 H_2; 347.1 H_2-18	**4.4%**	
	H_5N_3	**742.0**	**$[M+2Na]^{2+}$**	**18.4**	1298.5 H_4N_3; 1280.3/651.6 H_4N_3-18; 1257.4/640.2 H_5N_2; 1239.4 H_5N_2-18;1095.4 H_4N_2; 1077.4/550.2 H_4N_2-18; 933.4 H_3N_2; 694.4 H_3N-2x18; 568.4 H_2N; 550.2 H_2N-18; 509.0 H_3-18; 451.0 H_3N_2-3x9; 388.1 HN-18; 329.0 H_2-2x18; 226.0 N-18	**0.3%**	
	H_2S	**656.5**<br>**678.5**	**$[M+Na]^+$**<br>**$[M–H+2Na]^+$**	**12.8**		**0.4%**	
				**21.8**	516.3 HS; 498.2 HS-18; 480.1 HS-2x18; 365.0 H_2; 354.1/332.1 S; 336.0/313.9 S-18; 317.9/296.0 S-2x18	**1.7%**	
	HF	**349.2**	**$[M+Na]^+$**	**5.9**		**1.7%**	
	H_2F	**511.4**	**$[M+Na]^+$**	**7.0**	365.1 H_2; 349.1 HF; 347.1 H_2-18; 331.1 HF-18; 203.0 H; 185.0 H-18	**2.8%**	**Fuc{Gal(β1-4)Glc [37,46,47]**
	NF	**390.2**	**$[M+Na]^+$**	**6.5**		**0.3%**	
	HNF	**552.5**	**$[M+Na]^+$**	**6.7**	406.1 HN;388.2 HN-18; 349.1 HF; 243.9 N; 226.1 N-18; 203.0 H	**0.0%**	
				**7.0**	406.1 HN; 390.2 NF; 388.2 HN-18; 372.0 NF-18; 226.0 N-18; 203.0 H	**1.2%**	**GalNAc(α1-3)[Fuc(α1-2)]Gal [37]**
	H_2NF	**714.5**	**$[M+Na]^+$**	**6.7**	568.3 H_2N; 552.1 HNF; 550.4 H_2N-18; 534.3 HNF-18; 406.1 HN; 388.2 HN-18; 372.1 NF-18; 226.1 N-18; 208.0 N-2x18; 203.0 H	**1.1%**	
